# Supplementary material for: Central terminals of primary afferents coordinate the spontaneous activity of dorsal horn neurons
Source: J Physiol. 2025 Jun 7;603(12):3589–603. doi: 10.1113/JP287970 (PMC12206439; doi:10.1113/JP287970)
Supplement: Supplementary file 2 — Supporting Information [file TJP-603-3589-s001.pdf]

<u>**Control values for the whole set of observations**</u>

Frequency of spontaneous DRP (Hz)	Mean Amplitude of spontaneous DRP (µV)	Frequency of Population Bursts (Hz)	Mean Amplitude of Population Bursts (Hz)
0.195	19.822	0.256	74.952
0.102	14.149	0.03	40.419
0.152	10.009	0.365	33.546
0.403	14.791	0.165	62.918
0.329	9.227	0.383	49.23
0.03	9.521	0.289	98.712
0.242	10.478	0.329	81.622
0.367	10.661	0.134	127.078
0.189	10.626	0.224	96.255
0.413	13.492	0.113	94.465
0.238	10.847	0.344	123.187
0.513	12.971	0.236	144.342
0.608	14.714	0.284	130.791
0.210	28.577	0.199	36.566
0.270	14.832	0.229	50.47
0.303	8.974	0.175	100.609
0.377	20.436	0.234	46.974
0.316	19.908	0.225	74.87
0.398	13.193	0.396	116.579
0.076	13.194	0.132	64.373
0.234	12.662	0.129	58.259
0.142	12.021	0.121	91.008
0.267	12.554	0.148	109.092
0.100	12.304	0.193	92.031
0.047	8.757	0.104	76.012
0.479	12.745	0.518	83.603
0.298	12.474	0.087	36.585
0.293	9.438	0.132	46.355
0.359	30.416	0.115	108.401
0.37	15.546	0.225	147.152
0.434	26.852	0.361	98.989
0.085	33.381	0.043	30.261
0.398	11.788	0.354	22.126
0.713	26.334	0.643	31.124
0.099	12.334	0.169	153.12
0.483	13.712	0.483	102.913
0.384	16.919	0.203	44.471
0.449	8.828	0.368	46.361

Data for figure 3

Figure 3A

Irradiance (mW/cm^2)	Change in dorsal root basal potential (mV)												
1.0	0.044	0.023	0.018	0.008	0.022	0.037	0.035	0.046	0.048	0.050	0.013	0.027	0.009
1.5	0.098	0.071	0.099	0.072	0.065	0.065	0.128	0.120	0.138	0.124	0.030	0.083	0.047
3.2	0.144	0.099	0.152	0.105	0.093	0.082	0.198	0.185	0.211	0.192	0.044	0.106	0.102
4.5	0.179	0.135	0.187	0.143	0.116	0.092	0.235	0.227	0.268	0.233	0.050	0.124	0.160
12.6	0.232	0.219	0.289	0.278	0.172	0.127	0.298	0.296	0.349	0.310	0.060	0.168	0.325
31.0	0.279	0.265	0.395	0.337	0.203	0.155	0.311	0.333	0.389	0.347	0.062	0.196	0.424
42.3	0.315	0.268	0.420	0.339	0.209	0.159	0.312	0.328	0.392	0.342	0.063	0.202	0.445

Figure 3B

Irradiance (mW/cm^2)	Mean Firing Frequency per track (Hz)												
No light	0.504	0.480	0.343	0.653	0.685	0.814	1.078	0.222	0.655	0.549	0.552	0.598	1.433
1.0	0.432	0.479	0.415	0.620	0.673	1.316	1.356	0.284	0.737	0.548	0.481	0.750	1.524
1.5	0.480	0.448	0.393	0.717	0.690	1.640	2.109	0.581	1.190	0.839	0.499	1.879	2.217
3.2	0.587	1.040	0.649	0.955	1.095	1.824	2.418	1.129	1.709	1.104	0.585	2.619	2.904
4.5	0.586	0.964	0.679	1.233	1.263	1.966	2.588	1.538	2.027	1.345	0.854	2.710	3.208
12.6	0.830	0.918	0.902	1.547	1.725	1.901	3.158	2.103	2.523	1.468	1.150	2.764	3.723
31.0	0.797	0.763	0.979	1.648	1.723	2.032	3.408	2.452	2.840	1.489	1.079	2.920	4.166
42.3	0.740	0.969	0.866	1.560	1.854	2.040	3.215	2.476	2.882	1.472	1.249	3.011	4.144

Total No. of Neurons in track	70	21	35	46	33	75	64	70	59	61	69	84	86

Figure 3C

Irradiance (mW/cm^2)	Frequency of spontaneous DRP (Hz)												
No light	0.195	0.102	0.152	0.403	0.329	0.030	0.242	0.367	0.189	0.413	0.238	0.513	0.608
1.0	0.000	0.000	0.019	0.075	0.094	0.030	0.000	0.019	0.037	0.038	0.151	0.094	0.301
1.5	0.019	0.000	0.019	0.000	0.019	0.000	0.038	0.000	0.019	0.038	0.038	0.075	0.132
3.2	0.000	0.000	0.019	0.000	0.056	0.000	0.038	0.037	0.000	0.000	0.019	0.019	0.019
4.5	0.000	0.019	0.019	0.000	0.038	0.000	0.000	0.094	0.000	0.038	0.038	0.038	0.000
12.6	0.000	0.037	0.019	0.000	0.038	0.000	0.113	0.000	0.019	0.038	0.019	0.019	0.000
31.0	0.000	0.038	0.000	0.000	0.000	0.019	0.019	0.000	0.000	0.000	0.019	0.000	0.000
42.3	0.000	0.000	0.000	0.038	0.000	0.000	0.019	0.019	0.038	0.019	0.019	0.038	0.000

Figure 3D

Irradiance (mW/cm^2)	Frequency of Population Bursts (Hz)												
No light	0.256	0.030	0.365	0.165	0.383	0.289	0.329	0.134	0.224	0.113	0.344	0.236	0.284
1.0	0.218	0.019	0.282	0.094	0.189	0.224	0.321	0.076	0.187	0.075	0.282	0.169	0.301
1.5	0.302	0.038	0.339	0.113	0.094	0.114	0.151	0.075	0.170	0.113	0.282	0.132	0.113
3.2	0.263	0.000	0.225	0.132	0.188	0.056	0.075	0.019	0.057	0.075	0.189	0.000	0.057
4.5	0.189	0.056	0.207	0.188	0.132	0.000	0.019	0.000	0.038	0.019	0.132	0.000	0.019
12.6	0.000	0.019	0.113	0.038	0.038	0.000	0.000	0.000	0.000	0.000	0.094	0.000	0.000
31.0	0.019	0.038	0.094	0.000	0.000	0.019	0.000	0.000	0.000	0.000	0.131	0.000	0.000
42.3	0.076	0.019	0.132	0.000	0.000	0.000	0.000	0.000	0.000	0.038	0.056	0.000	0.000

Data obtained from hM4Di-DREADD mice

Spontaneous activity

Frequency of spontaneous DRP (Hz)

control	CNO 10µM
0.434	0.145
0.085	0.046
0.398	0.022
0.713	0.003
0.099	0.035
0.483	0

Mean Amplitude of spontaneous DRP (µV)

control	CNO 10µM
26.852	11.385
33.381	26.008
11.788	8.928
26.334	12.259
12.334	8.7
13.712	0

Frequency of Population Bursts (Hz)

control	CNO 10µM
0.361	0.184
0.043	0.01
0.354	0.118
0.643	0.4
0.169	0.025
0.483	0.265

Mean Amplitude of Population Bursts (Hz)

control	CNO 10µM
98.989	39.334
30.261	20.619
22.126	10.117
31.124	8.959
153.12	136.102
102.913	36.692

Synaptic responses

Integrated area eDRP (µV/s)

Control	CNO 10µM
151.156	102.868
231.889	140.496
16.194	5.415
74.644	27.507
70.172	22.61
57.655	20.574

Synaptic responses of neurons recorded from hM4Di-DREADD mice

Net firing (No. Action potentials)

# Neuron	Control	CNO 10µM	# Neuron	Control	CNO 10µM	# Neuron	Control	CNO 10µM
1	16	27	65	0	0	129	0	0
2	20	6	66	2	0	130	0	0
3	0	0	67	3	2	131	2	1
4	-1	0	68	0	1	132	0	0
5	1	4	69	0	0	133	0	1
6	0	0	70	0	0	134	3	0
7	-3	9	71	0	0	135	16	5
8	24	4	72	2	0	136	6	15
9	4	1	73	1	0	137	2	0
10	17	6	74	1	0	138	9	1
11	9	4	75	0	0	139	0	0
12	0	0	76	-1	1	140	7	0
13	0	0	77	2	6	141	0	0
14	7	0	78	0	-1	142	0	0
15	19	15	79	2	1	143	0	0
16	0	0	80	-3	-4	144	0	0
17	1	6	81	8	-1	145	0	0
18	13	12	82	5	1	146	0	0
19	11	9	83	11	1	147	1	0
20	-1	0	84	0	0	148	11	1
21	0	0	85	-1	0	149	4	1
22	-2	0	86	1	0	150	0	0
23	0	0	87	2	-1	151	8	2
24	-1	0	88	0	0	152	0	0
25	1	1	89	3	3	153	4	2
26	-2	0	90	0	0	154	0	0
27	1	0	91	6	8	155	5	3
28	-1	1	92	0	0	156	0	0
29	0	0	93	9	7	157	8	2
30	0	0	94	3	0	158	2	0
31	0	0	95	12	4	159	5	5
32	0	0	96	0	0	160	0	0
33	0	0	97	0	0	161	2	0
34	1	0	98	1	1	162	1	0
35	-1	0	99	1	6	163	1	0
36	0	0	100	1	-1	164	13	4
37	0	0	101	1	-3	165	0	0
38	0	0	102	7	0	166	0	2
39	0	0	103	1	0	167	0	0
40	1	0	104	1	-1	168	5	3
41	0	0	105	1	0	169	1	4
42	1	-1	106	3	0	170	1	0
43	0	0	107	0	0	171	1	1
44	0	0	108	0	0	172	6	-6
45	1	0	109	4	0	173	0	0
46	0	0	110	0	0	174	2	5
47	0	0	111	4	0	175	0	1
48	0	0	112	5	1	176	16	0
49	0	0	113	0	0	177	1	0
50	0	0	114	0	0	178	7	2
51	0	0	115	3	0	179	0	0
52	0	0	116	3	1	180	5	1
53	-1	0	117	0	0	181	1	0
54	4	1	118	1	2	182	9	0
55	0	0	119	0	0	183	1	0
56	0	1	120	1	0	184	0	0
57	0	0	121	0	0	185	0	9
58	-6	0	122	13	8	186	1	0
59	0	0	123	0	0			
60	-2	0	124	12	1			
61	-1	0	125	6	4			
62	-4	-2	126	5	0			
63	2	3	127	0	0			
64	3	0	128	0	0			

Data for figure 6

Figure 6A

Irradiance (mW/cm^2)	Change in dorsal root basal potential (mV)											
30,5	-0,194	-0,024	-0,012	-0,034	-0,079	-0,05	-0,168	-0,069	-0,073	-0,112	-0,079	-0,085
113,5	-0,301	-0,179	-0,039	-0,153	-0,145	-0,136	-0,259	-0,161	-0,169	-0,233	-0,237	-0,158
169,4	-0,396	-0,202	-0,049	-0,211	-0,176	-0,14	-0,287	-0,177	-0,193	-0,275	-0,284	-0,18
187,2	-0,31	-0,173	-0,069	-0,21	-0,18	-0,188	-0,298	-0,204	-0,219	-0,272	-0,296	-0,189

Figure 6B

Irradiance (mW/cm^2)	Mean Firing Frequency per track (Hz)											
No light	0,424	0,311	0,651	0,257	1,675	0,775	0,241	0,958	1,147	0,717	0,821	0,394
30,5	0,619	0,38	0,83	0,396	1,834	0,939	0,568	1,286	1,14	0,779	0,783	0,696
113,5	0,686	0,459	1,101	0,612	1,749	1,441	0,797	1,449	1,33	0,977	0,935	0,792
169,4	0,775	0,745	0,992	0,681	1,81	1,339	0,578	1,648	1,732	1,321	0,913	0,655
187,2	0,872	0,909	1,408	0,819	1,952	1,518	0,834	1,738	1,496	1,189	0,802	0,901

Total No. of Neurons in track	24	26	30	29	24	54	41	25	43	39	40	35

Figure 6C

Irradiance (mW/cm^2)	Frequency of spontaneous DRP (Hz)											
No light	0,210	0,270	0,303	0,377	0,316	0,398	0,076	0,234	0,142	0,267	0,100	0,047
30,5	0,425	0,377	0,377	0,343	0,321	0,397	0,341	0,356	0,265	0,285	0,113	0,246
113,5	0,301	0,338	0,378	0,375	0,320	0,471	0,282	0,397	0,359	0,367	0,245	0,283
169,4	0,416	0,475	0,417	0,471	0,322	0,454	0,263	0,378	0,358	0,453	0,188	0,226
187,2	0,416	0,360	0,460	0,472	0,379	0,451	0,283	0,428	0,378	0,472	0,207	0,302

Figure 6D

Irradiance (mW/cm^2)	Frequency of Population Bursts (Hz)											
No light	0,199	0,229	0,175	0,234	0,225	0,396	0,132	0,129	0,121	0,148	0,193	0,104
30,5	0,29	0,245	0,283	0,362	0,226	0,416	0,227	0,3	0,265	0,19	0,34	0,283
113,5	0,282	0,263	0,321	0,394	0,282	0,434	0,282	0,265	0,321	0,238	0,264	0,245
169,4	0,341	0,38	0,36	0,358	0,265	0,472	0,207	0,283	0,321	0,226	0,301	0,207
187,2	0,34	0,341	0,307	0,378	0,303	0,394	0,245	0,261	0,264	0,245	0,264	0,189

Figure 6E

Irradiance (mW/cm^2)	Mean Amplitude of spontaneous DRP (µV)											
No light	28,577	14,832	8,974	20,436	19,908	13,193	13,194	12,662	12,021	12,554	12,304	8,757
30,5	46,419	23,937	15,457	48,963	40,147	27,973	68,548	42,703	50,659	50,885	28,092	37,875
113,5	114,604	49,165	40,947	124,758	74,499	66,98	177,444	74,576	93,54	97,257	96,966	71,298
169,4	146,844	59,361	51,84	165,489	117,067	97,704	183,58	100,112	152,347	102,86	143,549	86,548
187,2	139,456	77,918	61,68	178,351	108,137	105,085	230,635	93,339	128,497	91,756	122,855	76,121

Figure 6F

Irradiance (mW/cm^2)	Mean Amplitude of Population Bursts (Hz)											
No light	36,566	50,47	100,609	46,974	74,87	116,579	64,373	58,259	91,008	109,092	92,031	76,012
30,5	127,911	84,836	152,346	68,583	83,952	178,933	148,191	85,21	130,124	132,324	96,987	111,362
113,5	150,256	92,556	188,828	141,024	129,055	322,73	203,285	122,183	156,123	166,383	170,911	132,737
169,4	152,822	115,682	208,433	198,25	167,158	301,14	186,169	137,935	196,796	234,235	135,715	137,865
187,2	173,028	105,288	286,812	191,468	163,291	386,266	171,568	180,927	162,585	189,268	132,405	158,56
